# Causes of Mortality of Dairy Cattle Diagnosed by Complete Necropsy

**DOI:** 10.3390/ani12213001

**Published:** 2022-11-01

**Authors:** David J. Wilson, Emma Jane Kelly, Savannah Gucwa

**Affiliations:** Animal Dairy and Veterinary Sciences Department, Utah State University, 4815 Old Main Hill, Logan, UT 84322, USA

**Keywords:** necropsy, dairy cattle, dairy calves, mortality, death, disease, diagnosis

## Abstract

**Simple Summary:**

Necropsy of dairy cattle is an important diagnostic tool, often more definitive or differing from the perceptions of dairy farm personnel in diagnosing the cause of death. This retrospective case series summarized the primary causes of death in all dairy animals diagnosed at necropsy from Utah and other states, mostly in the Intermountain West of the U.S. Some fatal diseases in dairy cattle that might be expected to be diagnosed ante-mortem were detected. Necropsy diagnosis is a guide to changes in management or preventive practices to reduce the rate of deaths in dairy herds.

**Abstract:**

This retrospective case series summarized the primary causes of death in 857 dairy cattle necropsied from 2008 to 2019 at the Utah Veterinary Diagnostic Laboratory, from dairy farms in Utah (76%), Idaho (16%) or other states (8%), primarily in the U.S. Intermountain West. Of cattle with age provided, 74% matched with body weight based estimates for those with no age stated. Cattle ranged from fetuses at 60 days of gestation to 9 years old. Primary cause of mortality was diagnosed in 833 cattle (97%); no cause was evident in 24 cattle (3%). Sexes were female 620 (72%), male 214 (25%), not recorded 23 (3%). Seven diseases killed 80% of the animals: gastrointestinal disease (most enteritis/colitis) 318 (37%); pneumonia 166 (19%); abortion 96 (11%), peritonitis 30 (4%), omphalophlebitis (navel ill) 27 (3%), abomasitis 23 (3%), and metritis 23 (3%). Etiologic agents and specific causes varied with age categories of the animals. Young calves that died from dystocia, omphalophlebitis, or congenital abnormality often presented with no suspicion of those causes by the owners because of no external signs. Some important fatal diseases of adult dairy cows that are often diagnosed ante-mortem were diagnosed at necropsy with no suspicion by those submitting the carcasses: metritis, hardware disease, and displaced abomasum. Multicentric lymphoma was a relatively important cause of death in cows more than 4 years old. Despite use of a toxicology laboratory, toxicity was only diagnosed as causing 1% of the deaths across all ages of dairy cattle. There were numerous other causes of mortality diagnosed as well. Necropsy is a vital tool to diagnose causes of death in dairy cattle and can guide changes in management or preventive practices to reduce the rate of deaths in dairy herds.

## 1. Introduction

Necropsy of dairy cattle is an important diagnostic tool, often more definitive than or differing from the perceptions of dairy farm personnel in diagnosing the cause of death [1,2,3]. Causes of dairy cattle death in the U.S. based on complete necropsy diagnoses have not been summarized in more than 50 years. Reports of dairy cattle necropsy results, primarily from outside of the U.S., have focused on certain age groups, including aborted fetuses [4,5], young calves [3,5] and lactating cows [2]. Whether by necropsy, or often based on questionnaires asking the opinions of dairy personnel on causes of animal deaths, many bovine mortality summaries focus on general categories of disease such as diarrhea/enteritis [6,7], respiratory disease [8,9], or mastitis [10,11].

The objective of this retrospective case series study was to summarize the primary cause of death in all dairy animals ranging in age from aborted fetuses to adult cows diagnosed at necropsy in Utah and other states, mostly in the Intermountain West of the U.S. This can assist in differential diagnoses and preventive management measures as well.

## 2. Materials and Methods

### 2.1. Study Population of Necropsied Animals

All dairy animals necropsied from 2008 to 2019 at the Utah Veterinary Diagnostic Laboratory (UVDL), including the main laboratory in Logan, UT and the branch laboratory in Spanish Fork, UT were included in the study. Animals submitted were selected entirely by the owners or with the advice of their veterinarians; sometimes the history provided included details of how many animals had died or been sick with similar signs on the farm recently, but the majority of cases did not arrive with such history. History, breed and age were obtained from the owner or submitter of the animal at the time of submission, but sometimes this information was unknown to the submitter. When no age was provided, age was estimated from body weight. Gestational ages of fetuses were estimated based on crown-rump lengths measured at necropsy.

### 2.2. Necropsy Sample Collection and Diagnostic Procedures

In accordance with accreditation by the American Association of Veterinary Laboratory Diagnosticians, there are Standard Operating Procedures for necropsies at the UVDL. During the time of the study, 6 pathologists and 4 residents under their supervision performed the necropsies using the standard procedures. Five of the 6 pathologists and eventually all 4 of the residents were board certified by the American College of Veterinary Pathologists.

For all diagnostic tests that the attending pathologists found indicated immediately at necropsy (e.g., bacterial culture, polymerase chain reaction-based [PCR] molecular testing, virus isolation, hepatic mineral analyses), fresh specimens were submitted for testing, sometimes refrigerated at 4 °C until testing began on the next work day. Samples collected from necropsied cattle differed between fetuses and postnatal cattle. For fetuses, abomasal fluid and tissue samples from kidney, liver, lung, and spleen were collected and stored at −20 °C for potential subsequent testing, Tissues from abomasum, adrenal gland, brain, heart, kidney, liver, lung, rumen, spleen, thymus, thyroid, and placenta (if submitted) were collected from fetuses and placed in 10% neutral buffered formalin (NBF) for histopathologic examination.

For postnatal cattle, fresh specimens were handled as described above. Tissue samples from kidney, liver, lung, and spleen were collected and stored at −20 °C for potential subsequent testing. Tissues from abomasum, adrenal gland, brain, duodenum, esophagus, heart, ileum, jejunum, kidney, lung, liver, lymph node, omasum, pancreas, reproductive organs (ovaries, uterus, testes), reticulum, rumen, skeletal muscle, spleen, trachea, thymus (juveniles), thyroid, and urinary bladder were collected and placed in 10% NBF for histopathologic examination. When indicated based on the animal’s history (if available) and observations at necropsy, additional tissues were collected. After fixation, samples for histopathologic examination were subsampled, processed, sectioned, and stained using hematoxylin and eosin. Special stains (e.g., Gram stain, periodic acid-Schiff, Giemsa, Masson-trichome) were applied to replicate sections in accordance with the judgement of the pathologist. Additional testing was performed when indicated, however when testing that necessitated additional charges to owners above the basic necropsy charge was offered, some owners declined the additional testing because of cost.

Bacterial infection was detected by histopathologic observation and/or microbiology (microbial diagnosis was often declined when the cost would have exceeded the basic necropsy fee). Tissue samples or fluids were inoculated onto Trypticase soy agar with 5% sheep blood, chocolate blood agar, Columbia nutrient agar, and MacConkey agar (all agars from Hardy Diagnostics, Santa Maria, CA, USA) for bacterial isolation. Culture plates were placed in a standard non-CO_2_ incubator at 37 °C for 24 h. Plates were examined for bacterial growth at 24 and 48 h. Resulting colonies were identified to genus or species level using either API (bioMérieux Inc., Durham, NC, USA) or BBL Crystal (Becton Dickenson and Co., Franklin Lakes, NJ, USA) test kits, or matrix assisted laser desorption ionization-time of flight mass spectrometry (MALDI-TOF, Bruker Daltronik GmbH, Bremen, Germany); MALDI-TOF was available beginning in 2014. Parasitism was detected by gross or histopathologic observation, polymerase chain reaction (PCR), or fecal flotation/sedimentation. Viral infection was diagnosed by enzyme-linked immunosorbent assay (ELISA), immunohistochemistry (IHC), PCR, or virus isolation. Hepatic mineral concentrations were quantified using inductively coupled plasma-mass spectroscopy (ICPMS). Vitamin E testing was performed at the California Animal Health and Food Safety Laboratory System (Davis, CA, USA).

### 2.3. Data Management and Diagnosis of Primary Cause of Death

Results of all necropsies were entered into a laboratory information management system (Vetstar Animal Disease and Diagnostic Systems, Advanced Technology Corp., Ramsey, NJ, USA) at each UVDL branch. For this study, that data was also entered into a database (Excel, Microsoft, Redmond, WA, USA) for further summary and analysis. Cause of death was determined based on the animal’s history (if provided), gross and histopathologic lesions, and results of ancillary tests, according to the judgement of the pathologist. When multiple conditions were diagnosed, the primary cause of death was determined. All cases were reviewed with a veterinary pathologist and the primary cause of death was verified for this retrospective case series study.

This was not a planned experimental study. Therefore, no statistical analyses were performed. However, there were biologically significant interesting differences by sex for some causes of death within some age groups, and this was reported if so.

## 3. Results

There were 857 dairy cattle necropsied from 6 November 2008 through 4 June 2019 at the UVDL, originating from dairy farms in Utah (n = 651, 76%), Idaho (n = 137, 16%) or other states (n = 69, 8%), primarily in the Intermountain West of the U.S. No age was provided for 440 (58%) of the 760 postnatal cattle. Of the 320 cattle with age provided, 237 (74%) matched with the body weight estimates; most discrepancy between weight proxy and stated age was in 1 to 4 yr old cattle. Ages ranged from 60 days of gestation to 9 years old. There were 22 cattle (3%) with neither a body weight or age recorded; they were counted only in the overall totals of causes of death, but could not be assigned to any age group. For the 97 fetuses, mean and median age were 196 d and 200 d of gestation, respectively. For the 760 postnatal cattle, the mean age at death was 381 d (12.5 months), but the median age was only 35 d. Sexes were female (n = 620, 72%), male (n = 214, 25%), sex not recorded (n = 23, 3%). Primary cause of mortality was diagnosed in 833 cattle (97%), and no cause of death was evident in 24 cattle (3%). Most common breeds were Holstein (n = 749, 89%), Jersey (n = 73, 9%), and Brown Swiss (n = 8, 1%).

Across all ages of dairy cattle, 7 diseases killed 80% of the animals: gastrointestinal disease (n = 318, 37%, consisting of enteritis/colitis [n = 256], hardware disease [n = 22], rumenitis [n = 8], left displaced abomasum [LDA] [n = 7], right displaced abomasum [RDA] [n = 5], ruminal acidosis [n = 5], reticulitis [n = 3], abomasal dilation [n = 2], esophageal perforation [n = 2], and single cases of abomasal torsion, cecal torsion, glossitis, intestinal torsion, intussusception of ileum and colon, omasal ulceration, ruminal necrosis, and ruminal/reticular impaction); pneumonia (n = 166, 19%); abortion (n = 96, 11%), peritonitis (n = 30, 4%), omphalophlebitis (navel ill) (n = 27, 3%), abomasitis (n = 23, 3%), and metritis (n = 23, 3%). The complete list of all causes of dairy cattle deaths is in Table 1.

Of the 96 abortions, the main causes were bacterial (n = 20, 21%), protozoal (n = 14, 14%), congenital abnormality (n = 7, 7%; 6 of the 7 were female fetuses), and idiopathic (n = 46, 48%). One other full-term fetus died from dystocia without ever having breathed; the lungs had never inflated. Bacterial causes of abortion were *Escherichia coli* n = 5, *Campylobacter fetus* subsp. *fetus* n = 2, and single cases of *Bacillus licheniformis*, *Listeria monocytogenes*, and *Pasteurella multocida*; 10 other cases had bacteria observed as the cause of death but the fetuses were too necrotic for further microbiological diagnosis. All 14 protozoal abortifacients were *Neospora caninum*. Congenital causes of abortion were hyperplastic goiter n = 2, with single cases of bilateral renal pseudocyst (congenitally dilated kidneys), mandibular brachygnathism with multiple congenital abnormalities, patent ductus arteriosus with bilateral cardiac ventricular dilation, a cloned fetus with hydronephrosis with adrenocortical hypoplasia, and a cloned fetus with tracheal hypoplasia. All causes of fetal death are shown in Table 2.

There were 36 (4% of all deaths) neonatal calves 1 d to 5 d old, mean age 2 d. Most common mortality causes were enteritis/colitis (n = 10, 28%; 9 of the 10 were females), dystocia (n = 7, 19%), congenital abnormality (n = 6, 17%), omphalophlebitis (n = 4, 11%), and pneumonia (n = 2, 6%). Enteric pathogens were *E*. *coli* n = 5, *Cryptosporidium* spp. n = 4, and a single case of bovine rotavirus. Omphalophlebitis cases included 2 with *E*. *coli* isolated, and 2 others with bacteria observed but no microbial diagnostic procedures were performed. Both pneumonia cases died from the combination of *Mannheimia haemolytica* and *Trueperella pyogenes* infections. Congenital abnormality deaths were single cases of atresia jejuni, adrenal medullary dysplasia with hydronephrosis in a cloned calf, patent ductus arteriosus, patent foramen ovale (in a cloned calf, spiral colon hypoplasia, and ventricular septal defect. All causes of neonatal calf death are shown in Table 3.

Calves that died from 6 d to 55 d old-after neonate status to approximately weaning age-were the largest age group in the study, 340 calves (40% of all deaths), with a mean age of 17 d. Major causes of death were enteritis/colitis (n = 193, 57%), pneumonia (n = 51, 15%), abomasitis (n = 20, 6%), omphalophlebitis (n = 20, 6%; 12 of the 20 were males), peritonitis (n = 10, 3%), bloat (n = 3, 1%), congenital abnormality (n = 3, 1%), hepatitis (n = 3, 1%), neurologic disease (n = 3, 1%), omasitis (n = 3, 1%), reticulitis (n = 3, 1%), and rumenitis (n = 3, 1%). Pathogens detected from enteritis or colitis cases were *Cryptosporidium* spp. n = 91, bovine coronavirus n = 41, *E*. *coli* n = 28, bovine rotavirus n = 25, *Salmonella* spp. n = 21, bovine viral diarrhea virus (BVD) n = 20, and *Clostridium* spp. n = 4; some cases were determined to have died from a combination of 2 of the previous agents. Pneumonia pathogens causing mortality were *E*. *coli* n = 9 (all from aspiration of gastrointestinal tract contents), *P. multocida* n = 8, *M. haemolytica* n = 5, *Mycoplasma bovis* n = 4, and bovine respiratory syncytial virus (BRSV) n = 3; there were 15 cases where bacteria were observed in tissues but no further microbial testing was performed. Bacteria isolated from abomasitis were *Sarcina* spp. n = 10 and *Clostridium* spp. n = 6, of which 3 were speciated to *C. perfringens*, and single cases of BVD and *E. coli*. Omphalophlebitis pathogens were BVD n = 2, bovine coronavirus n = 2, *E. coli* n = 2, *Sarcina* spp. n = 2, and single cases of *M. bovis* and *Salmonella* spp. Most (80%) of the peritonitis cases were sequellae of abomasal perforation, and most were too necrotic for bacterial identification, but pathogens diagnosed in peritonitis cases were *Sarcina* spp. n = 2, and single isolates of bovine rotavirus and *E. coli*. Fatal congenital abnormalities were single cases of congenital chondrodystrophy with palatoschisis (cleft palate) and mandibular brachygnathism, ventricular septal defect, and a cloned calf with patent foramen ovale and palatoschisis. All hepatitis cases were bacterial infections; bacterial isolates were single cases of *Salmonella* sp. and *S. Dublin*. Neurologic diseases were cerebral hematoma, *E. coli* meningitis, and meningoencephalitis. Omasitis pathogens were single cases of BVD and *Salmonella* sp. Reticulitis pathogens were bovine rotavirus n = 2. All 3 rumenitis cases were caused by bacteria; the only case with a pathogen isolated had *Salmonella* sp. All causes of preweaned calf death are shown in Table 4.

There were 117 (14% of all deaths) calves 2 mo to 5 mo old, mean age 101 d. Most common causes of death were pneumonia (n = 59, 50%), enteritis/colitis (n = 23, 20%), bloat (n = 3, 3%), neurologic disease (n = 3, 3%), polioencephalomalacia (n = 3, 3%), ruminal acidosis from grain overload (n = 3, 3%), congenital abnormality (n = 2, 2%), mesenteric torsion (n = 2, 2%), peritonitis (n = 2, 2%), rumenitis (n = 2, 2%), and a single case of LDA in a 3 mo old calf (1%). Pathogens isolated from pneumonia were *P. multocida* n = 17, *M. haemolytica* n = 13, *Salmonella* spp. (all from aspiration of gastrointestinal tract contents) n = 10, *T. pyogenes* n = 8, BRSV n = 3, *Klebsiella pneumoniae* n = 3, and *Histophilus somni* n = 2. Enteric pathogens were *Salmonella* spp. n = 7, coccidia (*Eimeria bovis* or *E. zuernii*) n = 5, *Cryptosporidium* spp. n = 5, and single cases of *E*. *coli* and BVD. Neurologic diseases were single cases of bacterial meningitis with no further microbial diagnosis, meningoencephalitis caused by *M. haemolytica* with *T. pyogenes*, and pons and trigeminal nerve abscessation. Congenital abnormality deaths were from single cases of chondrodysplasia with multiple vertebral malformations of thoracic vertebrae, and a cloned calf with ventricular septal defect, right ventricular hypertrophy, with hydronephrosis. Peritonitis cases were an infection following surgical cannulation and an intestinal perforation of undetermined origin. All causes of death in calves 2 to 5 mo old are shown in Table 5.

Dairy cattle that died from 6 mo to 1 yr old were the smallest age group in the study, 39 calves (5% of all deaths), mean age 293 d. Most common mortality causes were pneumonia (n = 16, 41%; 14 of the 16 were females), enteritis/colitis (n = 7, 18%), bloat (n = 3, 8%), and toxicity (n = 3, 8%). There was also one animal killed by a gunshot wound, not as a means of euthanasia but by stray hunting shot. Pneumonia pathogens were *P. multocida* n = 6, *T. pyogenes* n = 4, *M. haemolytica* n = 2, *Salmonella* Dublin n = 2, and single cases of BVD and *H. somni*. Pathogens from enteritis or colitis were coccidia n = 6, BVD n = 2 (one death was caused by BVD with coccidia, and the other BVD case was a persistently infected calf that died at 11 mo old). Toxicity deaths were caused by ingestion of Japanese yew by 2 animals that died on the same farm on the same day, and one case of hemoglobinuria from onion ingestion, an animal from the same farm as a >1 yr old that also ingested onion and died on the same day. All causes of death in cattle 6 mo to 1 yr old are shown in Table 6.

There were 90 (11% of all deaths) from >1 yr to 4 yr old, mean age 2.8 yr. Of the animals with sex recorded, there were 87 females and 1 intact bull. Major causes of death were pneumonia (n = 16, 18%), metritis (n = 12, 13%), traumatic reticuloperitonitis (hardware disease) (n = 10, 11%), peritonitis (n = 7, 8%), hepatitis (n = 6, 7%), displaced abomasum (n = 4 [2 left, 2 right displaced], 4%), hemorrhage (n = 4, 4%), toxicity (n = 4, 4%), enteritis/colitis (n = 3, 3%), mastitis (n = 3, 3%), and trauma (n = 3, 3%). Pathogens causing pneumonia were *M. haemolytica* n = 5, *P. multocida* n = 3 (one *P. multocida* case was also infected with BVD and BRSV), and single cases with *Citrobacter freundii*, *Pasteurella* sp., and *Streptococcus* sp. Metritis deaths were caused by *E. coli* n *=* 5, *Salmonella agona* n = 3, and one case with *T. pyogenes*. Peritonitis cases were caused by intestinal perforation n = 3 (one from an LDA-entrapped bowel loop, others idiopathic perforation), abomasal perforation n = 2 (one gravel ingestion, one perforated ulcer), one colonic perforated ulcer, and one peritonitis case with no evident cause. Fatal hepatitis case etiologies were hepatic abscess n = 3, hepatic lipidosis n = 2 and a bacterial hepatitis caused by *Salmonella* sp. Hemorrhage deaths resulted from single cases of bleeding abomasal ulcer, ruptured ovarian cystadenoma, jejunal hemorrhage, and uterine artery rupture. Three cases of toxicity were in 2 yr old cows that ingested water hemlock and died suddenly on the same farm on the same day; the other death was caused by hemoglobinuria from onion ingestion by a >1 yr old bull from the same farm as the onion toxicity case in a younger animal described earlier. Enteritis or colitis deaths were caused by single cases of *E. coli*, *Clostridium* sp., and *Mycobacterium avium* ssp. *paratuberculosis* (Johne’s disease). There was only one mastitis death where the milk was suitable for microbiological testing, caused by *Staphylococcus chromogenes* in an 18 month old heifer who had not yet calved. Trauma causing mortality included traumatic pharyngitis n = 2 (suspected as iatrogenic from oral administration of medications) and one case where the origin of traumatic injuries was not clear, with no history provided. All causes of death in cattle > 1 yr to 4 yr old are shown in Table 7.

There were 116 (4% of all deaths) from >4 yr to 9 yr old, mean age 5.9 yr. All 116 were cows. Most common causes of mortality were pneumonia (n = 18, 16%), hardware disease (n = 11, 9%), metritis (n = 11, 9%), enteritis/colitis (n = 10, 9%), peritonitis (n = 10, 9%), lymphoma (n = 9, 8%), displaced abomasum (n = 7 [4 left, 3 right displaced], 6%), bloat (n = 5, 4%), hemorrhage (n = 5, 4%), mastitis (n = 4, 3%), and hydroallantois (n = 3, 3%). Pathogens isolated from pneumonia were *M. haemolytica* n = 6, *P. multocida* n = 3, *E. coli* n = 2, and single cases of *Proteus* sp. and *T. pyogenes*. Metritis pathogens were *E. coli* n = 6, and one case of *Enterococcus* sp. There were 2 other cases of metritis caused by retained placenta; the submitters were unaware of the retained fetal membranes. Enteric pathogens were *Clostridium* spp. n = 4 (2 were further speciated as *C. perfringens*) and single cases of coccidia and *Salmonella* sp. Peritonitis deaths were caused by abomasal perforation n = 4 (one was following closed abdominal “roll and tack” surgery to attempt correction of an LDA), iatrogenic following cannula placement surgery n = 2, and single cases of rupture of the duodenum, ruptured intraabdominal abscess, and ruptured liver abscess, and a case with undifferentiated bacterial infection. All 9 cases of lymphoma were multicentric. Hemorrhage resulted from jejunal hemorrhage n = 3, and single cases of pulmonary hemorrhage and uterine artery rupture. Mastitis pathogens isolated were single cases of *Clostridium* sp., *Enterobacter* sp., and *E. coli*. All causes of death in cattle > 4 yr to 9 yr old are shown in Table 8.

## 4. Discussion

Gastrointestinal disease, primarily enteritis and colitis, and respiratory disease made up most of the causes of overall dairy cattle mortality diagnosed at necropsy, accounting for half of all deaths (30% and 20%, respectively). This agrees with the experience of the authors as well as previous reports of causes of dairy cattle deaths [1,2].

The proportion of deaths attributed to these diseases varied with age. During the first 2 months of life, calves died primarily of enteritis and/or colitis, followed by pneumonia, similar to previous reports of deaths in young dairy calves [3,5,12]. Interestingly, enteritis or colitis causing death of dairy calves often presents with no history from the submitters, and sometimes with no visible evidence of fecal soiling on the carcass at the beginning of necropsy [3]. Lack of external evidence in calves dying of enteritis with internal diarrheal fecal contents has been observed in some calves at necropsy by the authors as well. Among all calves during the first 2 months of life, *Cryptosporidium* spp. were the most common cause of enteritis deaths, followed by bovine coronavirus, *E*. *coli*, bovine rotavirus, *Salmonella* spp., and BVD. These results are similar to studies of enteric pathogens in dairy calves with diarrhea early in life, including reports on calves that lived as well as regarding calves that died [3,6,13,14].

Respiratory agents causing the most pneumonia deaths during the first 2 months of life were *P. multocida*, *M. haemolytica*, *M. bovis*, and *E. coli* from aspiration pneumonia. These are similar to respiratory pathogens reported previously in young dairy calves that recovered, with no examinations of dead calves, with the exception of *E. coli*, which some studies have identified as one of the important respiratory pathogens while others have not [15,16]. However, reports of etiologic agents detected at necropsy of dairy calves that died during the first 2 months of life from pneumonia are not found in the literature.

Other important causes of mortality in young dairy calves were dystocia, congenital abnormalities (separate from those that caused abortion of fetuses in utero), and omphalophlebitis in calves 1 to 5 days old, and abomasitis and omphalophlebitis in calves from 6 days to 2 months old. All of the above diseases caused between 6% and 20% of calf deaths until weaning age. Interestingly, fatal trauma from dystocia was not apparent to those submitting the calves for necropsy. Dystocia-induced trauma leading to death of calves in the first days of life has been reported previously [3,17,18].

Many calves submitted for necropsy with congenital abnormalities had visible defects, and were cloned calves from Utah State University’s cloning research programs, while a few were presented by owners concerned about valued genetic lines in their herds, including sale of animals for superior genetics in cow and bull families. However, some internal congenital abnormalities were not readily apparent until necropsy. The most common fatal congenital defects whether alone or in combination were cardiac abnormalities-ventricular septal defect and patent foramen ovale-or palatoschisis. This predominance of cardiac defects agrees with a previous report, but no summaries or descriptions of bovine congenital abnormalities have been published for over 35 years [19]. A single case report described palatoschisis in one Holstein calf [20].

Omphalophlebitis has also been observed as one of the major causes of death in dairy calves during the first month of life in other reports [21,22,23]. However, there are no previous reports of pathogens isolated from dairy calf omphalophlebitis as were characterized in the present study. Abomasitis was reported to cause 2.5% of the deaths in young beef calves [24], lower than in the present study of dairy calves, but there are no published summary data on abomasitis mortality among dairy animals. The lack of published reports may contribute to the fact that the authors have communicated with experienced members of the dairy industry who are not familiar with abomasitis as a cause of death in young dairy calves. Nevertheless, it was an important cause of early life mortality in this study. The most common bacterial isolates from abomasitis were *Sarcina* spp. and *Clostridium* spp., the latter being *C. perfringens* in all clostridial isolates that were speciated. These were the predominant pathogens in fatal abomasitis of young dairy calves in one previous case report [25], but another report of 23 dairy calves dying of abomasitis, many less than 3 weeks old, only found clostridial pathogens [26].

From 2 months to 1 year old, pneumonia emerged as the single most common cause of death, with enteritis becoming the second most common. This agrees with previous reports of causes of death as well as the importance of pneumonia in post-weaned calves that survived, but there is little data on pneumonia as a proportion of deaths in this age group [5,27,28,29]. Predominant respiratory pathogens causing death in animals from 2 months to 1 year old were *P. multocida*, *M. haemolytica*, and *T. pyogenes*. *Pasteurella multocida* and *M. haemolytica* have been cited as the most common causes of pneumonia including fatal cases in this age group [30,31], but *T. pyogenes* was diagnosed as the primary cause of death in the present study more frequently than some reports. However, a literature review found the latter to be an important respiratory pathogen in dairy cattle as well [32].

Other common causes of overall deaths across the dairy cattle population of all ages were abortion, peritonitis and omphalophlebitis (the latter was discussed regarding death of young calves above). Most abortions that were diagnosed were caused by bacterial (primarily *E. coli* or *C. fetus* subsp. *fetus* when a specific bacterial diagnosis could be made, but often no specific bacterial identification could be made) or protozoal (*N. caninum*) infections. There were fewer abortions caused by congenital abnormalities, more common in cloned calves; nearly half of abortions were idiopathic. Similar findings were reported in other studies of bovine abortions [4,5,33,34].

Nearly all peritonitis deaths were in preweaned calves up to 2 months old or in cows from 3 to 9 years old. From the few calf peritonitis necropsies where a pathogen was identified, *Sarcina* spp., bovine rotavirus, and *E. coli* were the etiologic agents. There are no refereed publications regarding causative pathogens of dairy calf peritonitis, although the disease is sometimes described as one of the important causes of calf death, albeit far less common than enteritis or pneumonia [3,21]. In adult cows, peritonitis resulted from perforation of various organs in the gastrointestinal tract, including perforated ulcers, ruptured abscesses, or iatrogenic following surgery. Ruptured liver abscesses and perforated ulcers associated with dairy cow peritonitis have been described previously [35,36], but their importance as a cause of dairy cattle deaths has not.

Among all dairy cattle more than 1 year old in the present study, pneumonia remained the most common cause of mortality while enteric disease was eclipsed by other categories of disease. Most of the literature regarding fatal pneumonia cases in cattle is about beef cattle, especially those in feedlots, rather than dairy cattle. Earlier reports of dairy cow mortality have found pneumonia as either the most common cause of death or one of the most common causes [1,2,30]. By far the most common pathogens causing fatal adult cow pneumonia in the present study were *M. haemolytica* and *P. multocida*, with numerous other pathogens being diagnosed as the primary cause only in single cases. Previous studies have also found these two agents to be the most common causes of pneumonia mortality in dairy cows, although some used molecular diagnostic methods that would not detect many of the other pathogens if present [30,37].

The next tier of relatively common causes of death in adult dairy cows included a group of diseases that are often diagnosed in live animals, and even if fatal, might intuitively be expected to have been diagnosed on the farm rather than the animals being submitted for necropsy. These diseases were metritis, traumatic reticuloperitonitis (hardware disease), LDA and RDA. Metritis has been addressed in several previous studies but was not reported as a cause of dairy cattle death; rather, it was described as causing reproductive failure [1,2,38]. Most metritis cases were caused by *E. coli*, in agreement with previous reports of metritis pathogens in live cattle [39,40]. Some fatal metritis infections had pure isolates of *S. agona*, which has increasingly been isolated from “diseased” dairy cattle in recent years [41], but has not been reported as a cause of bovine metritis. Hardware disease has only been reported as an important cause of death diagnosed at necropsy in beef cows, with similar results to the present study [42], but a large case series study of mostly Holstein cattle reported that 12% to 29% of animals with hardware were euthanized, and that not all cattle with hardware can be diagnosed antemortem [43]. Displaced abomasa, both LDA and RDA, caused 5% of all of the unknown deaths in adult cows and in one 3-month-old calf submitted for necropsy, and this was excluding other deaths from complications of attempted surgical correction. It was surprising that these displacements were not evident in live cattle, thus resulting in submission of the carcasses to diagnose the cause of death. Auscultation of live animals, use of confirmatory ultrasonography (uncommon but growing in use), or a gross necropsy on farm soon after death would diagnose the disease [44]. While there are no previous refereed publications regarding the proportion of dairy cattle deaths caused by untreated abomasal displacements, the prognosis for uncorrected cases such as those reported here is poor. One study following 672 cows found that 79% of cows with untreated LDAs died [45]. One of the authors, DW, has observed an LDA in another young dairy calf; while rare, the disease has been reported in a 2 month old calf [46].

Only among cows more than 4 years old, multicentric lymphoma was a relatively important cause of mortality, 8%. This age range agrees with a summary of necropsies of cattle deaths from multicentric lymphoma [47], but the importance or proportion of total dairy cattle mortality caused by lymphoma has not been reported.

Despite the presence of a full-service toxicology laboratory at the veterinary diagnostic laboratory, and the relatively common suspicion of toxicity among livestock owners when animals die suddenly, toxicity was only diagnosed as the cause of approximately 1% of all deaths across all ages of dairy cattle. All were from ingestion of toxic plants: water hemlock, Japanese yew, or onion. These plants have all been described as highly toxic to cattle, often resulting in presenting signs of being found dead [48,49,50].

## 5. Conclusions

Three diseases killed 67% of all dairy cattle presented for necropsy, enteritis/colitis, pneumonia and abortion. Numerous different etiologic agents were isolated, varying with age of the animals. Young calves that died from trauma of dystocia, congenital abnormalities, or omphalophlebitis often presented with no suspicion of those causes by the owners because of no external signs. Some important fatal diseases in dairy cattle more than 1 year old might be expected to be diagnosed ante-mortem instead of being submitted for necropsy: metritis, hardware disease, LDA and RDA. Multicentric lymphoma was a relatively important cause of death in cows more than 4 years old, while despite use of a full-service toxicology laboratory, toxicity-from ingestion of water hemlock, Japanese yew, or onion-was only diagnosed as causing 1% of the deaths across all ages of dairy cattle. There were numerous other causes of mortality diagnosed as well. Necropsy is a vital tool to diagnose causes of death in dairy cattle and a guide to changes in management or preventive practices to reduce the rate of deaths in dairy herds.

## Figures and Tables

**Table 1:** Primary cause of death of 857 dairy cattle of all ages, including fetuses, in the Intermountain West diagnosed at necropsy from 2008–2019.

Primary Cause of Death	Number of Cases	% of all Deaths
Enteritis/Colitis	256	30.0
Pneumonia	166	19.4
Abortion	96	11.2
Peritonitis	30	3.5
Omphalophlebitis	27	3.2
Abomasitis	23	2.7
Metritis	23	2.7
Hardware Disease ^¥^	22	2.6
Bloat (Ruminal)	17	2.0
Liver Disease (Hepatitis)	15	1.8
Hemorrhage	12	1.4
Congenital Abnormality ^∞^	11	1.3
Emaciation	10	1.2
Multicentric Lymphoma	10	1.2
Dystocia	9	1.0
Rumenitis	8	0.9
Left Displaced Abomasum	7	0.8
Mastitis	7	0.8
Neurologic Disease	7	0.8
Toxicity	7	0.8
Trauma	6	0.7
Right Displaced Abomasum	5	0.6
Ruminal Acidosis	5	0.6
Septicemia	5	0.6
Omasitis	4	0.4
Mesenteric Torsion	4	0.4
Myositis	4	0.4
Bacillary Hemoglobinuria	3	0.4
Endocarditis, Vegetative	3	0.4
Hydroallantois	3	0.4
Polioencephalomalacia	3	0.4
Reticulitis	3	0.4
Abomasal Dilation	2	0.2
Esophageal Perforation	2	0.2
Laminitis	2	0.2
Abomasal Torsion	1	0.1
Arthritis (Polyarthritis)	1	0.1
Cecal Torsion	1	0.1
Cellulitis	1	0.1
Deficiency Copper, Iron, Selenium	1	0.1
Glossitis	1	0.1
Hematopoietic Sarcoma	1	0.1
Hemosiderosis	1	0.1
Intestinal Torsion	1	0.1
Intussusception of Ileum, Colon	1	0.1
Ketosis	1	0.1
Nephritis, Interstitial Ascending	1	0.1
Omasal Ulceration	1	0.1
Pleuritis	1	0.1
Ruminal Necrosis	1	0.1
Ruminal/Reticular Impaction	1	0.1
Idiopathic	24	2.8
TOTAL	857	100.0

^¥^ Hardware Disease = Traumatic reticuloperitonitis. ^∞^ Congenital abnormality = abnormality causing death of animals that lived until after birth; there were 7 other congenital cases of abortion (see Table 2).

**Table 2:** Primary cause of death of 97 dairy cattle fetuses diagnosed at necropsy.

Primary Cause of Death	Number of Cases	% of all Deaths
Abortion-Bacterial	20	20.6
Abortion-Protozoal (*N. caninum*)	14	14.4
Abortion-Congenital Abnormality	7	7.2
Abortion-Necrotic ^£^	6	6.2
Abortion-Infectious ^∞^	2	2.1
Abortion-Aspiration	1	1.0
Dystocia ^§^	1	1.0
Abortion-Idiopathic	46	47.5
TOTAL	97	100.0

^£^ Necrotic = Too decayed to attempt any diagnosis. ^∞^ Infectious = Infectious cause evident from pathology but too contaminated for further diagnosis. ^§^ Dystocia = Full term fetus with traumatic signs of dystocia, lungs never inflated.

**Table 3:** Primary cause of death of 36 neonatal (1 day to 5 days old) calves diagnosed at necropsy.

Primary Cause of Death	Number of Cases	% of all Deaths
Enteritis/Colitis	10	27.8
Dystocia	7	19.4
Congenital Abnormality	6	16.6
Omphalophlebitis	4	11.1
Pneumonia	2	5.5
Abomasal Dilation	1	2.8
Abomasal Torsion	1	2.8
Abomasitis	1	2.8
Bloat	1	2.8
Hemorrhage (Umbilical Artery) ^£^	1	2.8
Rumenitis	1	2.8
Idiopathic	1	2.8
TOTAL	36	100.0

^£^ Calf died on first day of life.

**Table 4:** Primary cause of death of 340 post-neonatal to approximate weaning age (6 days to 55 days old) calves diagnosed at necropsy.

Primary Cause of Death	Number of Cases	% of all Deaths
Enteritis/Colitis	193	56.8
Pneumonia	51	15.0
Abomasitis	20	5.8
Omphalophlebitis	20	5.8
Peritonitis	10	2.9
Emaciation	9	2.6
Bloat	3	0.9
Congenital Abnormality	3	0.9
Liver Disease (Hepatitis)	3	0.9
Neurologic Disease	3	0.9
Omasitis	3	0.9
Reticulitis	3	0.9
Rumenitis	3	0.9
Esophageal Perforation	2	0.6
Arthritis (Polyarthritis)	1	0.3
Dystocia	1	0.3
Glossitis	1	0.3
Intussusception of Ileum, Colon	1	0.3
Mesenteric Torsion	1	0.3
Omasal Ulceration	1	0.3
Pleuritis	1	0.3
Ruminal Acidosis	1	0.3
Ruminal/Reticular Impaction	1	0.3
Septicemia	1	0.3
Trauma ^¥^	1	0.3
Idiopathic	3	0.9
TOTAL	340	100.0

^¥^ Trauma = Bilateral femoral head luxation, hemorrhage; suspected from dystocia extraction but no history provided.

**Table 5:** Primary cause of death of 117 calves 2 months to 5 months old diagnosed at necropsy.

Primary Cause of Death	Number of Cases	% of all Deaths
Pneumonia	59	50.4
Enteritis/Colitis	23	19.6
Bloat	3	2.5
Neurologic Disease	3	2.5
Polioencephalomalacia	3	2.5
Ruminal Acidosis	3	2.5
Congenital Abnormality	2	1.7
Mesenteric Torsion	2	1.7
Peritonitis	2	1.7
Rumenitis	2	1.7
Emaciation	1	0.9
Endocarditis, Vegetative	1	0.9
Hemorrhage (Abomasal Ulceration)	1	0.9
Left Displaced Abomasum	1	0.9
Liver Disease (Hepatitis)	1	0.9
Myositis	1	0.9
Omphalophlebitis	1	0.9
Septicemia	1	0.9
Typhlitis	1	0.9
Idiopathic	6	5.1
TOTAL	117	100.0

**Table 6:** Primary cause of death of 39 calves 6 months to 1 year old diagnosed at necropsy.

Primary Cause of Death	Number of Cases	% of all Deaths
Pneumonia	16	41.0
Enteritis/Colitis	7	17.9
Bloat	3	7.6
Toxicity	3	7.6
Abomasitis	1	2.6
Bacillary Hemoglobinuria	1	2.6
Hardware Disease ^¥^	1	2.6
Hemorrhage (Pulmonary Artery)	1	2.6
Liver Disease (Hepatitis)	1	2.6
Mesenteric Torsion	1	2.6
Neurologic Disease	1	2.6
Trauma-Gunshot ^∞^	1	2.6
Idiopathic	2	5.1
TOTAL	39	100.0

^¥^ Hardware Disease = Traumatic reticuloperitonitis. ^∞^ Trauma-Gunshot = Stray hunting shot.

**Table 7:** Primary cause of death of 90 cattle > 1 year old to 4 years old diagnosed at necropsy.

Primary Cause of Death	Number of Cases	% of all Deaths
Pneumonia	16	17.9
Metritis	12	13.4
Hardware Disease ^¥^	10	11.1
Peritonitis	7	7.8
Liver Disease (Hepatitis)	6	6.7
Hemorrhage	4	4.5
Toxicity	4	4.5
Enteritis/Colitis	3	3.3
Mastitis	3	3.3
Trauma	3	3.3
Bloat	2	2.2
Left Displaced Abomasum	2	2.2
Right Displaced Abomasum	2	2.2
Myositis	2	2.2
Septicemia	2	2.2
Abomasal Dilation	1	1.1
Bacillary Hemoglobinuria	1	1.1
Cellulitis	1	1.1
Endocarditis, Vegetative	1	1.1
Hematopoietic Sarcoma	1	1.1
Hemosiderosis	1	1.1
Intestinal Torsion	1	1.1
Ketosis	1	1.1
Ruminal Acidosis	1	1.1
Idiopathic	3	3.3
TOTAL	90	100.0

^¥^ Hardware Disease = Traumatic reticuloperitonitis.

**Table 8:** Primary cause of death of 116 cattle > 4 years old (oldest 9 years old) diagnosed at necropsy.

Primary Cause of Death	Number of Cases	% of all Deaths
Pneumonia	18	15.5
Hardware Disease ^¥^	11	9.4
Metritis	11	9.4
Enteritis/Colitis	10	8.6
Peritonitis	10	8.6
Multicentric Lymphoma	9	7.8
Bloat	5	4.3
Hemorrhage	5	4.3
Left Displaced Abomasum	4	3.4
Mastitis	4	3.4
Hydroallantois	3	2.6
Right Displaced Abomasum	3	2.6
Laminitis	2	1.7
Liver Disease (Hepatitis)	2	1.7
Rumenitis	2	1.7
Abomasitis	1	0.9
Bacillary Hemoglobinuria	1	0.9
Cecal Torsion	1	0.9
Endocarditis, Vegetative	1	0.9
Myositis	1	0.9
Nephritis, Interstitial Ascending	1	0.9
Omasitis	1	0.9
Ruminal Necrosis	1	0.9
Septicemia	1	0.9
Trauma	1	0.9
Idiopathic	7	6.0
TOTAL	116	100.0

^¥^ Hardware Disease = Traumatic reticuloperitonitis.

## Data Availability

Data is available upon request from the corresponding author.
